# A Simple Platform for the Rapid Development of Antimicrobials

**DOI:** 10.1038/s41598-017-17941-7

**Published:** 2017-12-14

**Authors:** Stephen Albert Johnston, Valeriy Domenyuk, Nidhi Gupta, Milene Tavares Batista, John C. Lainson, Zhan-Gong Zhao, Joel F. Lusk, Andrey Loskutov, Zbigniew Cichacz, Phillip Stafford, Joseph Barten Legutki, Chris W. Diehnelt

**Affiliations:** 1 Biodesign Institute Center for Innovations in Medicine, Arizona State University, Tempe, Arizona 85281 United States; 2 Present Address: Caris Life Sciences, Phoenix, Arizona USA; 3 Present Address: Merz Aesthetics, Mesa, Arizona USA; 4 Present Address: School of Biological and Health Systems Engineering, ASU, Tempe, Arizona USA; 5 Present Address: Paradigm, Inc., Phoenix, Arizona USA; 6 Present Address: HealthTell, Inc., Chandler, Arizona USA

## Abstract

Recent infectious outbreaks highlight the need for platform technologies that can be quickly deployed to develop therapeutics needed to contain the outbreak. We present a simple concept for rapid development of new antimicrobials. The goal was to produce in as little as one week thousands of doses of an intervention for a new pathogen. We tested the feasibility of a system based on antimicrobial synbodies. The system involves creating an array of 100 peptides that have been selected for broad capability to bind and/or kill viruses and bacteria. The peptides are pre-screened for low cell toxicity prior to large scale synthesis. Any pathogen is then assayed on the chip to find peptides that bind or kill it. Peptides are combined in pairs as synbodies and further screened for activity and toxicity. The lead synbody can be quickly produced in large scale, with completion of the entire process in one week.

## Introduction

There is wide recognition of the need for the development of new antibiotics^1–3^. Historically, there has never been a wide selection of effective antivirals, with only antivirals for human immunodeficiency virus^4^, hepatitis B^5^, hepatitis C^6^, influenza^7^, herpes and cytomegalovirus^8^ available in the clinic. The deficiencies in the development pipeline have been magnified in outbreaks of new pathogens, such as for Severe Acute Respiratory Syndrome (SARS) and Middle East respiratory syndrome (MERS) caused by coronaviruses^9^, or the wider emergence of a known pathogen, such as the Ebola outbreak in West Africa^10–12^ and Zika virus in the Americas^13^. Particularly in the case of an emergency, it would be ideal to have a standard, ready-to-run platform for developing thousands of doses of a new antibiotic or antiviral against the emergent agent in a short time. If the new agents had a high probability of low toxicity and high efficacy it would decrease the time to their use in the emergency. Here we present a concept for developing antibiotics or antivirals in a systematic, potentially rapid manner based on the synbody technology and test its feasibility.

In designing a fast response system, we applied the following requirements. We assumed the infecting agent is isolated and available. It may not be required for it to be alive, relieving the necessity for high-level containment. We required that the creation of the therapeutic agent could be accomplished in 1 week or less with at least 1,000 doses produced. The production would integrate simultaneous toxicity screening to increase the probability of an approved therapeutic. We did not require that the antibiotic or antiviral be orally available as in an emergency intravenous administration may be adequate.

We used the synbody technology^14–21^ as the starting point for developing a platform to meet these specifications. Synbodies are bivalent peptides with antibody like features that are chemically synthesized. Two peptides that bind different regions of a chosen target, usually with low affinity and specificity, are linked to create a high affinity, high specificity reagent. The two arms of the synbody are chosen from a premade set of 10,000 peptides from random sequence space that are arrayed on slides. We felt the modular aspect of the synbodies might lend them to rapid production, particularly for a large number of doses in a short time. Additionally, the surface of viral and bacterial pathogens present repeating binding elements providing additional avidity between bivalent synbodies and targets on the surface of pathogens. Below we detail the concept and test the feasibility of its features to produce antibiotics and antivirals.

## Results

### System overview

**Figure 1:**
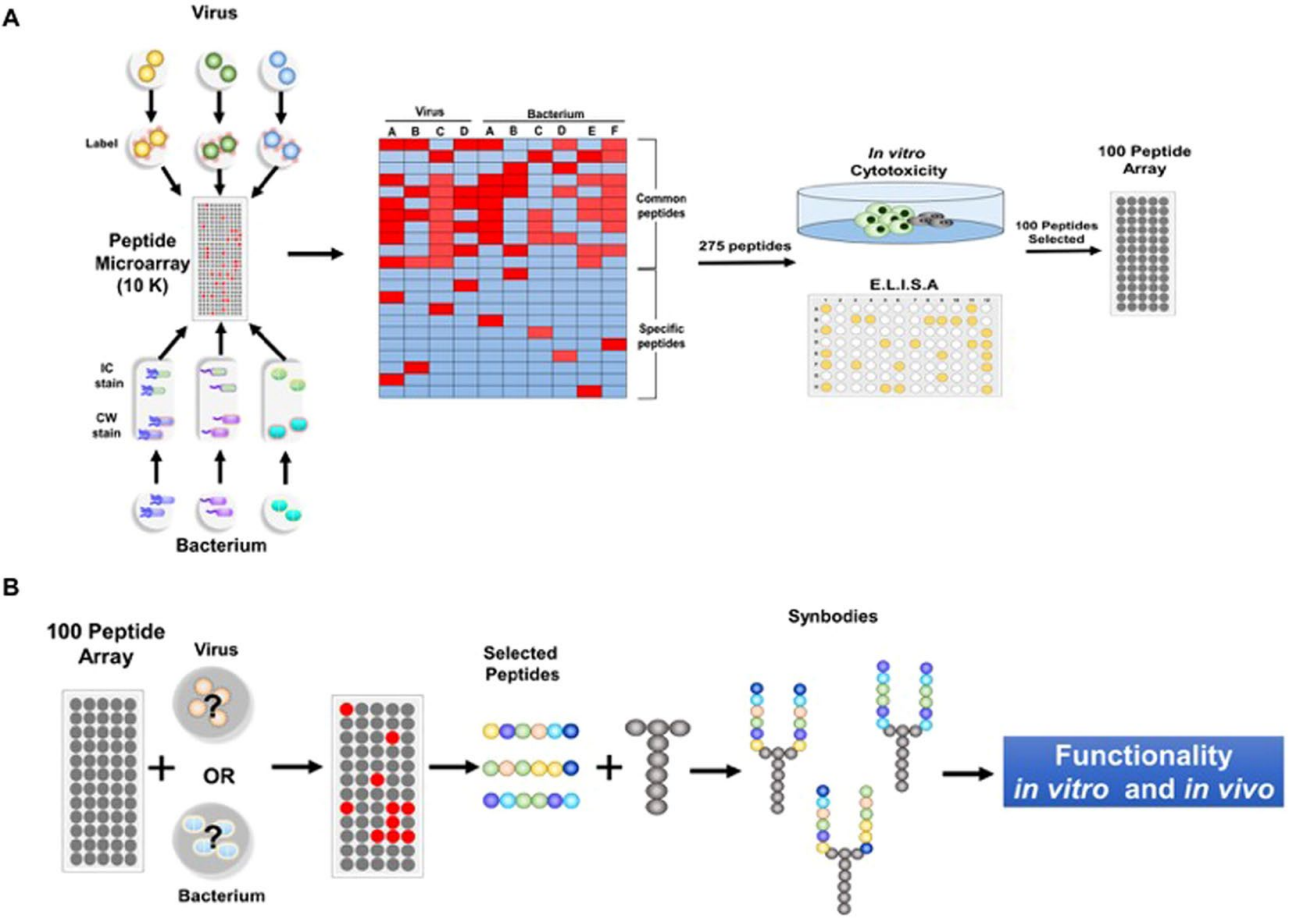
Development of pathogen binding 100-peptide microarray and rapid synbody discovery system. (**A**) A range of pathogens (10 viruses and 11 bacteria) were screened against a library of 10,000 peptides to identify shared and specific pathogen binding peptides. A total of 275 peptides were selected for secondary binding screening and down-selected for cellular toxicity. Peptides with confirmed binding and minimal toxicity were selected for inclusion into the pathogen binding peptide microarray. (**B**) Workflow for discovery of antimicrobial synbodies. A new or unknown pathogen is fluorescently labeled and screened against the pathogen binding 100-peptide microarray. Peptides that bind the pathogen are selected and conjugated to a synbody scaffold to produce a synbody library for activity and toxicity testing in a series of *in vitro* functional assays to select antimicrobial synbodies for additional development. IC stain: Intracellular staining; CW stain: Cell wall staining.

We have shown that bacteria^18^ and viruses^20^ can be applied to peptide microarrays to generate synbodies with antibiotic or antiviral activity. The challenge was to create a system to generate the synbodies quickly and provide sufficient quantities of the chosen synbody for *in vivo* testing. The key issue was that the published process involved applying the bacterial target to 10,000 peptide microarrays (10 K), choosing and testing target peptides, synthesis of large amounts of two or more candidate peptides, synthesis of synbodies and retesting. This process usually takes several months, with the rate-limiting step the synthesis and purification of large amounts of the candidate peptides. Our solution to this time issue was to pre-screen a large number of pathogens on the 10 K peptide microarray to arrive at 100 peptides that would offer sufficient diversity that any pathogen screened would bind two or more of peptides (Fig. 1A). By selecting peptides that are somewhat pathogen specific and others that are more broadly reactive, we should be able to select a reduced set of peptides with the potential to bind any new pathogen screened against these 100 peptides. It would then be practical to synthesize large stocks of these 100 peptides in advance so that 1,000 or more doses of a therapeutic could be produced quickly. Once the 100-peptide microarray was developed and the stocks synthesized it would be the starting point for the development of any therapeutic. As shown in Fig. 1B, a pathogen is incubated with the 100-peptide microarray and peptides binding it identified. These peptides are linked in all combinations to create lead synbodies, using the peptide and linker stocks. These leads are screened for activity against the target, preferably in blocking activity in an *in vitro* assay. Candidate synbodies are produced in large amounts, purified and tested in mice for acute toxicity and on red blood cells for hemolysis. The goal is to integrate the component steps so the whole process could be completed in 1 week.

### Creating the 100-peptide microarray

**Figure 2:**
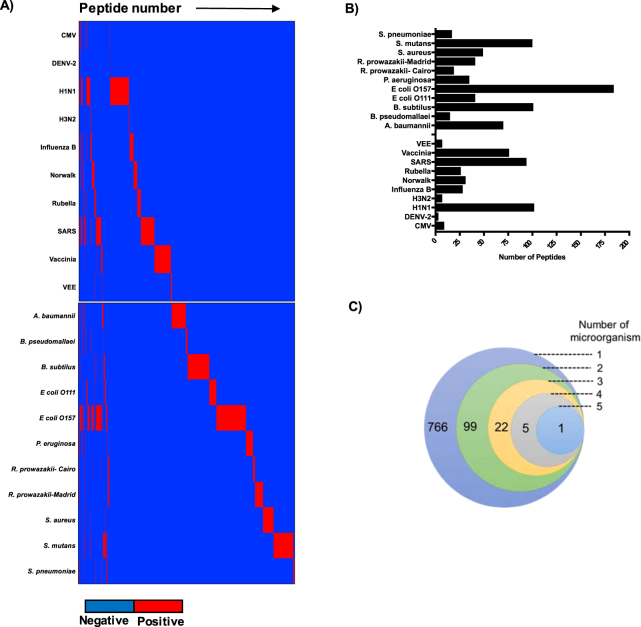
Bacterial and viral screening against 10,000 peptide microarrays identifies common and specific pathogen binding peptides. (**A**) Heat map of peptides (x-axis) that bound pathogens (y-axis). Positives peptides were defined as those with median normalized signal >2x background or >1.5x higher than the detection antibody control. For bacteria screened with the IC and CW labeling assay, positive peptides were positive in both fluorescent channels with a CW/IC ratio <5 (non-lytic peptides). Peptide intensities are colored in blue for negative and red for positive. (**B**) Total number of peptides positive for each pathogen evaluated. (**C**) Graphical representation of the number of common peptides shared amongst pathogens.

To create the 100-peptide library that could potentially bind any pathogen, we screened 21 different viruses and bacteria, representing a wide range of pathogenic bacteria and viruses on a peptide microarray (Supplementary Table S1). The 10 K array consists of 10,000 peptides spotted in duplicate on a standard size glass microscope slide with each peptide composed of 17 variable amino acids and an N-terminal CSG- constant region used for immobilization to the surface^22^. The slide surface is coated with a polymer to increase the peptide density and to reduce non-specific binding^18^. The peptide of each feature is of known sequence and the variable amino acid positions in the peptide are composed of 18 different amino acids with a slight for amino acids with aromatic side chains (Supplementary Fig. S1). The bacteria in the panel were screened for binding peptides in one of two ways. Live bacteria were screened using a previously published method^18^ while inactivated bacteria were detected using antibodies that were specific to that pathogen. In the live bacteria screening assay, the target is labeled with an amine-reactive dye, AlexaFluor (AF), and an internalizing dye, Cell Tracker Orange (CTO), and peptides that bind the bacteria without perturbing the membrane produce fluorescence in both channels while those that disrupt the membrane only produce fluorescence in the AF channel. Viruses were screened by detecting binding to a peptide using a fluorescently labeled virus-specific antibody, or in some cases directly labeling the virus with an Alexa Fluor dye^20^. Antibody detection was favored as it avoids the problems of the dye interfering with virus binding or binding the peptide itself. The microarray data was analyzed and binding peptides were selected according to the following criteria: (i) for pathogens detected via antibody, peptides with signals 1.5x higher the antibody only control; (ii) for direct labeled pathogens, peptides with signals 2x the background; or for live bacteria, peptides with AF/CTO <5. This selection strategy yielded a total of 893 peptides (Fig. 2).

A heat map of the data demonstrates that there are peptides that bind a single organism while there are those that bind multiple pathogens (Fig. 2A, Supplementary Information). Some organisms had relatively few peptide hits, while others such as *E. coli* O157:H7 had over 150 peptides that passed the selection criteria (Fig. 2B). The majority of peptides bound 1 pathogen, while 127 peptides bound more than 1 pathogen (Fig. 2C). From this data set, we selected the 127 multi-pathogen peptides and 161 single pathogen binding peptides for a total of 288 peptides that could maximally cover the pathogen space. These peptides were then synthesized in three 96-well plates and screened in secondary assays for binding and toxicity.

The 288 peptides were synthesized on the 5 mg scale with an N-terminal biotin and used without purification (Supplementary Information). When the library quality was analyzed by mass spectrometry, a small number of peptides (13 peptides) did not contain the full-length peptide at high abundance when analyzed by mass spectrometry and were not included in subsequent assays, reducing the library size to 275 peptides. The first issue was to confirm that the peptides bound the target pathogen previously used for selection.

#### Pathogen binding

**Figure 3:**
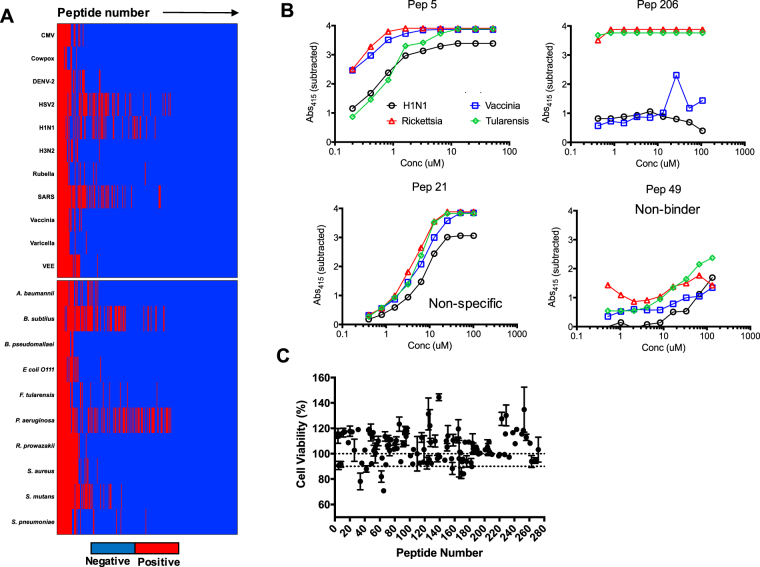
Secondary screening and evaluation of peptides for inclusion in the pathogen binding 100-peptide microarray. (**A**) Heat map of pathogens screened against 275 peptide array. Each pathogen was labeled with AF555 and AF647 and incubated on the array. (**B**) Representative binding curves from secondary screening of 275 peptide library by ELISA. 96-well plates were coated with Vaccinia virus (blue squares), A/PR/8/34 H1N1(black circles), *F. tularensis* (green diamonds), or *R. prowazekii* Madrid (red triangles) and each peptide was incubated at the indicated concentrations. (**C**) *In vitro* cytotoxicity screen of peptide library. HEK293 cells (1 × 10^6^ cells) were incubated for 1 hour with 25 µM of each peptide in replicate wells. Few peptides decreased cell viability >10% (dotted line).

Peptides must function when immobilized on a microarray, therefore we printed the 275 peptide candidates as a new microarray using the same methods as for the 10 K array. We then screened the 10 viruses and 11 bacteria (Supplementary Table S1) that had been previously tested on the 10 K array to verify binding to the appropriate pathogen. To increase confidence in the binding data, we changed the detection method for several bacterial pathogens, from AF-633/CTO labeling used in the 10 K array experiments to a dual membrane labeling approach using AF555 / AF647. Peptides that bound in both array detection methods were considered true hits. From this analysis, we found that 175 peptides showed binding for 1 or more pathogens (Fig. 3A, Supplementary Information).

Each peptide was then tested in an ELISA assay to determine its relative affinity for four different pathogens: vaccinia virus, H1N1 influenza virus, and Gram-negative pathogens *F. tularensis*, and *R. prowazekii*. Representative results from peptides binding to each pathogen are presented in Fig. 3B (Supplementary Fig. S2). As can be seen, some peptides showed high binding across all pathogens and all tested concentrations, indicating likely promiscuous or non-specific binding. However, other peptides had concentration dependent changes in pathogen binding. While absolute K_D_ values could not be determined with this method as unpurified peptides were used, relative K_D_ values could be estimated, with many peptides exhibiting concentration dependent binding. Peptides of this phenotype were classified as probable pathogen binders while non-specific binding or non-binding peptides were rejected. A total of 118 peptides passed this screening. In parallel with the binding assays, peptides were screened in an *in vitro* cytotoxicity assay using HEK293 cells. Peptides were screened at 0.25, 2.5 and 25 μM and the cell viability at 25 μM was plotted (Fig. 3C). Few peptides exhibited toxicity above the assay noise and those peptides with the highest toxicity were rejected. Based on these data the peptides were ranked by: (1) 275-peptide array binding (2) binding to 1 or more pathogens in ELISA and (3) by *in vitro* cytotoxicity. A total of 82 peptides showed binding in both binding assays and an additional 18 peptides showed binding in the peptide array only. Analysis of the peptides showed that there was a wide distribution of net charges and isoelectric points (Supplementary Fig. S3). Only 66% of this library is cationic in contrast to peptides in the antimicrobial peptide (AMP) database of which ~82% are cationic^23^. The 100 peptides were synthesized commercially (Sigma) at the 2-gram scale and purified to >95% purity (Supplementary Table S2). This stock was the source of peptides for printing 100 peptide arrays and for the synthesis of the synbodies.

**Figure 4:**
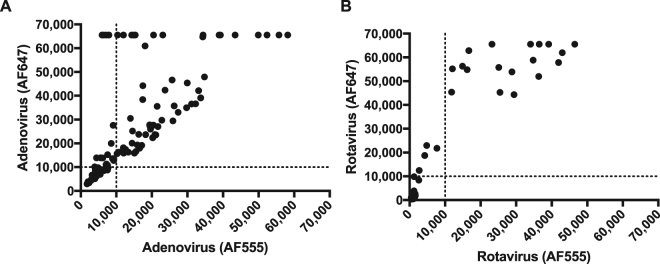
Evaluation of performance of the pathogen binding 100-peptide microarray. (**A**) Scatterplot of adenovirus binding to the 100-peptide microarray detected via AF555 (x-axis) or AF647 (y-axis). The data for each point is the average RFU across replicate peptide spots and microarrays. (**B**) Scatterplot of rotavirus binding to the 100-peptide microarray detected via AF555 or AF647.

To confirm that an unknown pathogen would bind to peptides from this reduced library, we screened two pathogens that were not used as part of the peptide selection process, adenovirus and rotavirus. Adenovirus is a non-enveloped, double-stranded DNA virus while rotavirus is a non-enveloped, double-stranded RNA virus. Each virus was labeled with AF555 and AF647 and tested using the same procedures as before. When the fluorescent signal across both channels is compared, it can be seen that multiple peptides bound each new pathogen (Fig. 4). There was correlation between both fluorescent dyes and binding peptides for a new pathogen could be easily identified. For example, peptides with relative fluorescence values >10,000 for both fluorophores could be selected as binding peptides for the new virus. In this way, multiple peptides could be selected for synbody construction. These data indicate that this array can potentially identify binding peptides for any given pathogen.

### Rapid Synbody Discovery

The creation of the 100-peptide microarray allowed implementation of the selection process. As shown in Fig. 1B, a pathogen of interest is applied to the microarray and peptides selected based on the relative florescence. Seven to 10 peptides are selected and the 100 peptide stock used to synthesize all combinations of the peptides into synbodies. We developed a modular and combinatorial approach using maleimide-thiol conjugation to rapidly produce synbody libraries^19–21^ where selected peptides are conjugated to different peptide scaffolds, each with two conjugation sites, in a combinatorial manner and in parallel. With this approach, dozens or even hundreds of synbodies can be produced in a day. The synbodies are evaluated for their potency, efficacy, and toxicity with the best selected for production in large scale. Once the synbodies are produced they can be screened for pathogen binding by ELISA, *in vitro* activity in either growth inhibition assays for antibacterial or in plaque reduction assays for antiviral synbodies, red blood cell toxicity and kidney toxicity. Candidates that have high target binding, *in vitro* activity and low toxicity are then prepared on large scale (>100 mg) with the same chemistry and purified by HPLC. Parallel primary screening, rapid synbody production using stocks of pre-made peptides, and parallel activity and toxicity testing enable this system to produce synbodies candidates in a very rapid fashion.

Once the basic component steps had been optimized a test run for a specific pathogen was executed to determine the time to complete synbody production. We chose A/CA/07/2009 H1N1 influenza, a major health concern worldwide as a viral test and *S. epidermidis*, which causes surgery related infections and is a source of antibiotic resistance genes, as a bacterial target.

### Development of an Antiviral

**Figure 5:**
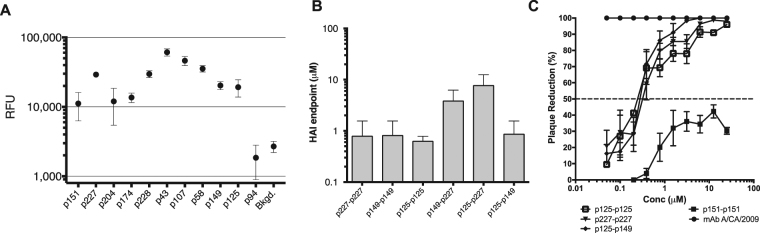
Development of antiviral synbodies against A/California/07/2009 pdm09 H1N1, a model unknown virus. (**A**) Relative fluorescence (RFU) for each peptide from binding of pdm09 H1N1. Values represent mean ± s.e.m. for six replicate spots per peptide. Background binding was measured in empty spots (Bkgd.) and peptide hits had mean binding that was significantly higher (p < 0.01) while a negative control peptide (p94) did not. (**B**) Inhibition of pdm09 H1N1 hemagglutination by synbodies. Error bars represent the standard error from replicate assays. (**C**) Plaque reduction of pdm09 H1N1 infected MDCK cells with candidate synbodies. A neutralizing pdm09 H1N1 mAb was used as a positive control and three HAI inhibiting (p125-p125, p125-p149, p227-p227) and a non-HAI inhibiting synbody (p151-p151) were tested. Error bars represent the standard error from replicate assays.

To test in principal whether an antiviral synbody could be produced by this protocol, we focused on development of a viral infection inhibitor for pandemic influenza (A/CA/07/2009 pdm09 H1N1). We screened H1N1 pdm09 on the 100-peptide array using the two color fluorescent labeling strategy. Based on the relative binding to pdm09 we selected 10 peptides (Fig. 5B) and tested these for the ability to bind hemagglutinin (HA) and inhibit hemagglutination by pdm09 of red blood cells with different concentrations (50 µM to 1.76 µM). Peptides p227, p149, and p125 inhibited hemagglutination. While p151, p204, p174, p228, p43, p107, and p58 bound A/CA/07/2009 on the peptide array, they did not prevent hemagglutination even in higher concentrations (50 µM) (data not shown). Based on HAI screening, peptides p227, p149, and p125 were used to prepare synbodies through pairwise conjugation to a previously developed synbody scaffold, Sc2^20^. Each synbody was hydrolyzed to prevent thiol-exchange reactions that occur when maleimide conjugated peptides are exposed to excess thiols, such as in albumin^19^. Synbodies were tested in the HAI assay and p227-p227, p125-p149, p125-p125, and p149-p149 each exhibited inhibition at 0.781 µM (Fig. 5B). Finally, to test if the synbodies prevented viral cell entry, synbodies p125-p125, p227-p227, p125-p149, and a negative control synbody p151-p151, were tested in a plaque reduction assay (Fig. 5C). Cells treated with the neutralizing antibody had 100% inhibition while the synbodies exhibited a concentration dependent decrease in plaque formation. The IC_50_ for p227-p227, p125-p149, and p125-p125 were 0.45 µM, 0.34 µM, and 0.31 µM respectively. These synbodies were then tested for hemolysis at 125 µM, a concentration roughly 100 times the IC_50_, and exhibited less than 10% hemolysis (Supplementary Fig. S4) indicating low red blood cell toxicity. These data indicate that a new virus can be screened against the small peptide library to discover binding peptides that can be converted into neutralizing synbodies in a rapid manner.

### Development of an Antibiotic

**Figure 6:**
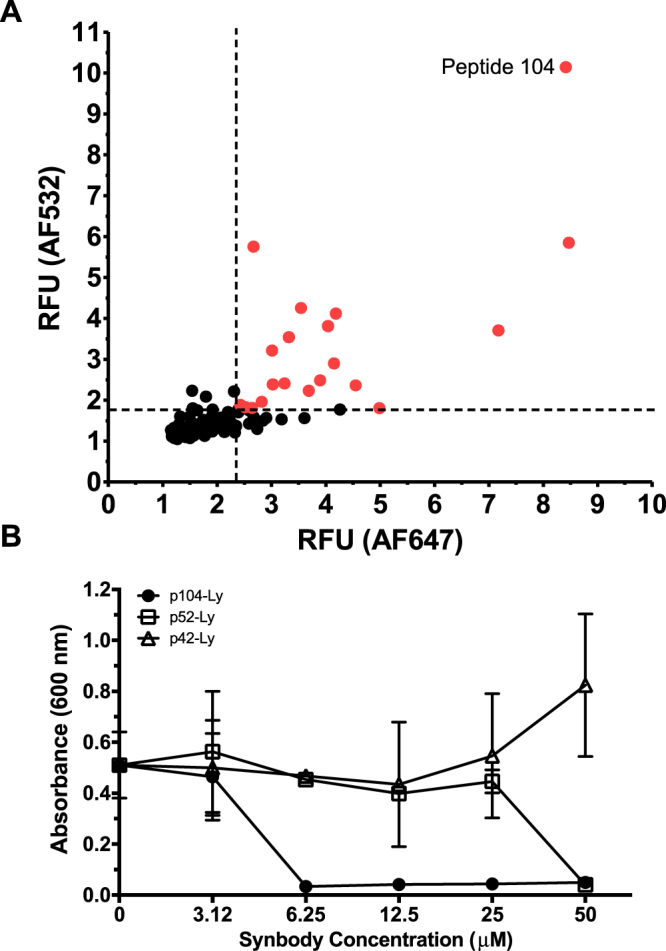
Development of antibacterial synbodies against *S*. *epidermidis*. (**A**) *S. epidermidis* was labeled with CTO and AF647 and 106 CFU/mL *S. epidermidis* was screened against the 100-peptide microarray. (**B**) Synbodies were tested for *S. epidermidis* growth inhibition after 18-hour treatment at the indicated concentrations.

The data presented above indicates that it is feasible to develop a substantial amount of a synbody anti-viral in one week. We then used the same system to produce a synbody with activity against a bacterium, *S. epidermidis*. While *S. epidermidis* is from the same genus as *S. aureus* used in the primary selection assay, there are large phenotypic differences between *S. epidermidis* and *S. aureus* as well as considerable intra strain variation^24^. As the peptides that were selected for the prototype 100 peptide microarray were designed to broadly bind pathogens rather than kill a specific bacteria, we used our previously demonstrated strategy of using a binding peptide conjugated to a killing peptide^18^ to produce synbodies for the proof-of-principle study. *S. epidermidis* was screened against the peptide array and 20 peptides selectively bound *S. epidermidis* but were predicted to have low killing activity based upon their AF/CTO ratio (Fig. 6A). These peptides were tested by MIC and they did not inhibit *S. epidermidis* growth at 50 uM (not shown). Therefore, we selected two positives binders (p52 and p104) and one negative control peptide (p42) and conjugated to an *S. aureus* lytic peptide^19^, called Ly, to produce a small synbody library that was then tested for inhibitory activity. We found that synbodies p42-Ly did not inhibit *S. epidermidis* growth at 50 uM, as expected, while p107-Ly, and p104-Ly inhibited bacterial growth with MICs of 12.5 µM, and 6.25 µM, respectively (Fig. 6B). Synbodies were then tested for hemolysis at 125 uM and no hemolysis was observed (Supplementary Fig. S3). These data indicate that the synbody system can also be used to quickly produce antibacterial candidates for additional development.

## Discussion

We described a strategy to employ the synbody platform to enable the creation of 1000 s of doses of a potential antibiotic or antiviral in a week. We first developed a 100-peptide microarray, with peptides that would bind one or more pathogens. To do this we screened 21 diverse bacteria and viruses on a 10,000 peptide microarray. Over 1% of the peptides on the array bound multiple pathogens, with some lytic to bacteria, and these were chosen for further screening. These peptides were synthesized and assessed for binding the pathogen panel by ELISA-type binding and for *in vitro* toxicity to human cells. A library of 100 peptides were chosen, synthesized and purified on large scale. These peptides were printed on small microarrays and were the core for the rapid screening. A system for rapidly synthesizing and screening synbodies was demonstrated. As a demonstration project synbodies against influenza virus that is effective *in vitro* were produced and the same process was used to produce an antibiotic against *S. epidermis* in one week which inhibited growth at 6.25 µM.

We had previously demonstrated that synbodies could be developed with antibiotic activity^18^ and shown the value of using D amino acid-containing peptides^19,20^. The challenge we addressed here was whether the process could be done very quickly. The key was developing the 100-peptide microarray that was representative enough of broad pathogen-binding/lytic space. This allowed us to pre-stock large amounts of peptides for combinatorial production of the synbodies. Of note, the cost of synthesis of peptides has decreased notably and economies of scale enable the commercial production of numerous peptide therapeutics^25^. The second new feature was the development of a system for the rapid synthesis and screening of candidate synbodies. This required the optimization of the conjugation chemistry. The third new aspect was the incorporation of rapid toxicity screening.

An underlying assumption of this approach was that pathogen surface space has enough redundancy that 100 different peptides could cover its diversity. That this was true implies that pathogens as diverse as bacteria and viruses have overlapping chemical diversity. All the pathogens screened were infectious agents. It is possible that being a human pathogen and evolving against the human immune systems constrains the surface diversity of any pathogen. Analysis of the 100 peptide sequences did not reveal any obvious trends or common motifs that would suggest targeting a specific pathogen through homology with a natural receptor. Alternatively, the 100 peptides are predicted to lack secondary structure given the random amino acid distribution and length, and therefore could bind other targets with comparable affinity but through a different mechanism of action. We are currently investigating this further.

The demand on time did not allow us to incorporate one synbody related technology. We have shown that a candidate peptide could be greatly improved in affinity or refined in other selectable features^16^. This was done by making a matrix of single amino acid variants and screening them. The increase in affinity was simply additive for the variants. However, relative to this process the addition of this step would add at least 9 weeks (largely due to time to synthesis and the iterative nature of the optimization method) to the time required. This may be an acceptable addition in most circumstances of emergency but we wished to test the limits of the process in this demonstration.

As an example, we outline how this process as described might be applied to a situation like the 2014 Ebola outbreak in West Africa. The first step would be to apply intact Ebola virus to the 100-peptide array. This could be done using high titer inactivated blood or sera in a BSL2 facility. The virus binding to peptides would be detected using labeled anti-Ebola antibody with the total assay time of less than 1 day. This process would identify ~10 peptides that would be used to create a panel of synbodies. Conjugation reactions of synbodies would be performed in parallel and proceed to completion within a few hours. Purification of synbodies is performed by HPLC in parallel and these synbodies could be screened for binding inactivated Ebola virus in a BSL2 facility. Cell toxicity and hemolysis screening could be done in parallel. Positive candidates would then have to be screened for inactivation of virus in a cell-based assay that would require BSL4 containment. Two or more of the best candidates could then be synthesized from the large stocks and purified. Presumably, these synbodies could then be tested in animal models for safety and protection in challenge models.

This system will have several limitations. Though even dangerous pathogens can be screened in BSL2 facilities if killed, the activity screens would have to be conducted with live pathogen at higher containment. Screening with killed or viable viruses probably will not matter. However, a screen with killed bacteria would not discriminate lytic peptides. In this case it may require attaching a bacterial killing element to the binding synbody. For example, we, and others, have explored using ligands to recruit existing antibodies to pathogens^26–28^. Finally, the synbodies as produced by this process would probably not be orally administrable unless further formulated. However, they may be used by intravenous injection. Possibly the biggest concern for employing this system is that the product is a peptide-based therapeutic. While antimicrobial peptides (AMPs) have a poor track record in producing clinical products^29^ antibiotics such as daptomycin, vancomycin, and colistin are peptidic. Additionally, the clinically approved HIV fusion inhibitor enfuvirtide is a 36-aa peptide with an elimination half-life of 3.8 hours^30^. There are many standard methods for improving the protease stability of peptide therapeutics, including the incorporation of D-amino acids^19,20^, and would be used in future implementations of the system.

We demonstrated a potential workflow to produce 1000 s of doses of an anti-infective. To produce enough product for a population-wide use would require substantial scaling in production of peptides and purification of the synbodies. However, even the focused, rapid use of an anti-infective could be useful. For example, in the Ebola outbreak, the protection of healthcare workers was a major concern and for the Zika outbreak the concern is for women who might become pregnant.

We consider synbody antibiotics and antivirals to have more similarity to antibody therapeutics than AMPs. Antibodies and serum therapy have been used as both antiviral and antibacterial treatments with a number of monoclonal and polyclonal antibodies approved^31^. Some have argued that antibodies should be revisited as a mode of treating infections, particularly in light of increased antibiotic resistance^32^. Clearly, cost would be a major concern for the broad use of antibodies. In this regard, synbodies may have an advantage in that they should be less expensive to chemically synthesize than biologically produced antibodies. A unique feature of the use of the peptide synbody scaffold is that the same platform can produce synbodies with direct antibiotic or antiviral activity and should be useful for primary and secondary infections.

In summary, we have demonstrated the feasibility of producing candidate antibiotics or antivirals in a very short time. With a scaled pre-investment, even a wider selection of peptide candidates could be used on the selection microarray and more doses of each candidate made. Some of the features demonstrated here, for example the rapid synthesis of a large number of synbody candidates, could also be used in standard approaches to therapeutic development. Alternatively, parallel advances in high density peptide microarray synthesis^33–38^ and rapid peptide synthesis^39^, could dramatically increase the diversity of the peptide screening and decrease time to synthesize the resulting hits. These emerging technologies could eliminate the need for library pre-synthesis and yet maintain the speed of the discovery platform, increasing the potential for this approach.

## Methods

### Peptide Microarrays

Glass microscope slides are coated with polyethylenimine prior to peptide printing in order to create a high density peptide surface in each feature^18^. Sulfosuccinimidyl 4-(N-maleimidomethyl)cyclohexane-1-carboxylate (Sulfo-SMCC) is applied to the amine surface to activate the microarray prior to printing the peptides. The peptides are 20 amino acids long with a CSG linker on the N-terminus, with the N-terminal Cys functioning as the conjugation site to the peptide microarray. Cysteine is excluded from the 17aa variable region. The peptide sequences were generated from a random number generator, with minor amino acid biases. Peptides were synthesized by Sigma Custom Peptide (The Woodlands, TX) and used without purification. Peptides were diluted to 1 mg/mL in 25 μM 4-(2-hydroxyethyl)-1-piperazineethanesulfonic acid (HEPES) pH 7.3 + 10% acetonitrile (AcCN) prior to dispensing. Peptides were printed by Applied Microarray, Inc. (Tempe, AZ) using non-contact piezo printing in 90% humidity. Arrays were washed in 70% AcCN, 30% methanol (MeOH), then washed in water (ddH_2_O) for 1 hour prior to use.

### Peptide Microarray Screening

Peptide microarrays were blocked with 600 μL blocking buffer [3% bovine serum albumin (BSA), 0.05% Tween20, 0.134 mg/ml Mercaptohexanol in 1x Tris buffered saline (TBS)] in a humidity chamber for 1 hour at room temperature. Microarrays were washed with washing buffer (1x TBS + 0.05% Tween20) followed by two washes with ddH_2_O. Virus solutions were prepared in dilution buffer (5 mg/mL BSA + protease inhibitor), applied to the peptide microarray, and incubated in Agilent microarray chambers for 1 hour at 37 °C. Microarrays were washed, probed with 250 μL of the appropriate detection antibody in Agilent microarray chambers for 1 hour at 37 °C, washed and probed with 250 μL of 5 nM AlexaFluor-647 conjugated secondary antibody for 1 hour at 37 °C. Arrays were washed, dried, and scanned on an Agilent Microarray Scanner. Each sample was run on duplicate arrays. Each primary antibody was screened in absence of pathogen as a negative control. Data were analyzed with Microsoft Excel or JMP Pro 13. For bacteria binding we used our previously published method^18^.

### Synbody Library Production

Synbodies were produced according to developed methods^19–21^. Synbody scaffolds were synthesized by Sigma Custom Peptide at 90% purity and used as before. Briefly, the peptide and the scaffold are separately dissolved in 30% AcCN. One equivalent of the scaffold is mixed with two equivalents of the peptide; the pH of the reaction mixture is adjusted to 6.5–7.0 with the addition of dilute 10% trimethylamine (TEA) in AcCN. The reaction incubates at room temperature and was monitored by HPLC and MALDI-MS. Synbodies are purified by HPLC, molecular weight confirmed by MALDI, and lyophilized prior to use.

### Peptide ELISA Assay

Nunc MaxiSorp flat bottom 96 well ELISA plates were coated with 50 μL of 0.2 μg/well of each pathogen in ELISA coating buffer and kept overnight at 4 °C. Plates were washed three times with 1x phosphate buffered saline + 0.05% Tween20 (PBST) using a BioTek ELx405 plate washer followed by blocking with 100 μL 3% BSA in 1x PBST for 2 hours at 37 °C. Plates were washed and biotin labeled peptides added in dilution buffer [0.1% BSA + 1x PBST + 0.05% v/v Tween20] and incubated for 1 hour at 37 °C. Plates were washed, 100 μL of 1:2000 streptavidin-Horseradish peroxidase (HRP) was added, and incubated for 1 hour at 37 °C. Plates were washed, 100 μL of 3,3′,5,5′-tetramethylbenzidine (TMB) (Thermo Scientific) was added, and incubated in the dark for 15 minutes at room temperature. The reaction was quenched with 100 μL of 0.5 M hydrochloric acid (HCl) and read immediately at 450 nm using a microplate reader (Spectra MAX 190, Molecular Devices, Inc.). Peptides that exhibited concentration dependent binding, were screened again at appropriate concentrations to confirm pathogen binding.

### Measurement of Cytotoxic Potential of Peptides

HEK293 cells were washed in assay medium, diluted to the desired concentrations, and dispensed at 100 μL in a sterile 96-well tissue culture plate with 50,000 cells added in each well. The cells were incubated overnight (37 °C, 5% CO2, 90% humidity). In order to remove residual LDH activity from the cells, the overnight assay media was replaced with new media. Peptides were diluted to 2 times their final concentration in assay media and dispensed at 200 μL in a separate 96-well plate. Peptides were then dispensed at 100 μL into duplicate or triplicate wells of the cell containing plate along with the appropriate controls and incubated for 24 hours (37 °C, 5% CO2, 90% humidity). Supernatant was carefully removed and transferred into an optically clear 96-well flat bottom microplate where freshly prepared LDH reaction mixture (Roche) was dispensed at 100 μL in each well and incubated for 30 min at 25 °C. To stop the reaction, 50 μL of stop solution was added to each well and the absorbance of the samples were measured using a microplate reader (Spectra MAX 190, Molecular Devices, Inc.). Cytotoxicity was calculated according to the manufacturer’s recommendation and cell viability from replicate wells was calculated as 1 – cytotoxicity.

### Hemagglutination Assay

Initial hemagglutination assays were done to determine the titer to be used for subsequent hemagglutination inhibition and were repeated for each new turkey red blood cell (tRBC) lot. Initially 50 µL of 1x PBS solution was added to all wells to be used in a 96 plate. The initial titer for A/CA/7/2009 H1N1 pdm09 (BEI Resources: NR-13663) was added in duplicate at concentrations of 1/50 in 50 µL. The virus was serially diluted in 2 fold dilutions across the plate. 50 µL of a solution of 0.5% tRBCs (Innovative Research cat no. IR1-110N) were added, and the plate was incubated at 30–60 minutes at room temperature. After incubation the plate was checked for hemagglutination. A positive hemagglutination result was determined by the appearance of a diffuse red color across the well. A negative result appeared as dots in the center of the round bottom plates. The HA titer was determined as the highest dilution factor to cause a positive hemagglutination result. This minimum HA concentration was labeled as 1 HAU unit. Subsequent testing of new lots of tRBC’s followed the same procedure as above except with starting virus concentration of 1/25.

### Hemagglutination Inhibition Assay

For the hemagglutination inhibition (HAI) assay, the viral titer previously determined was used. For the initial assay of candidate peptides 25 µL of PBS was added to all wells of a 96-well plate. For each peptide, 25 µL of 200 µM solution and 25 µL of PBS was added prior to serial dilution across the row in 2 fold dilutions. Twenty-five µL of a 1/100 dilution of pdm09 was added to all of the wells to achieve a concentration of 1 HAU. The plate was incubated for 30 minutes at room temperature. Then, 50 µL of 0.5% tRBC’s were added and the plate was incubated for 30–60 minutes. The hemagglutination was then measured. The minimum inhibitory concentration for each peptide was defined as the highest concentration that produced a negative hemagglutination result. Subsequent assays were run as before starting with 25 µM or 50 µM of synbody that was 2-fold diluted across the row.

### Plaque Reduction Neutralization Assay

Madin-Darby canine kidney cells (MDCK) were grown in 6-well plates (1 × 10^6^ cells/well). Then, pdm09 influenza (100 PFU) was incubated with serial dilutions (50 µM to 0.05 µM) of synbodies in minimal essential medium (MEM) (Gibco) for 1 hour at 37 °C. Mixtures were transferred onto MDCK cells in 6-well plates and incubated 1 hour at 37 °C with 5% CO_2_. After incubation, virus and synbody mixtures were removed and cells were overlaid with 0.3% agarose-MEM medium supplemented with 1 μg/ml of TPCK-trypsin (Sigma). Infected MDCK cells were cultured for 2 days 37 °C at 5% CO_2_, fixed with 3% formaldehyde and stained with 1% crystal violet. Number of plaque forming unit was determined and percentage of plaque reduction was calculated based on the PFU produced by the virus without treatment. An anti-pdm09 monoclonal antibody (serial dilutions from 1:100 to 1:25,600) and p151-p151 synbody were used as positive and negative controls. The monoclonal Anti-Influenza Virus antibody was obtained through BEI Resources, NIAID, NIH: Monoclonal Anti-Influenza Virus H1 Hemagglutinin (HA), A/California/04/2009 (H1N1) pdm09, Clone 4F8 (produced *in vitro*), NR-42021. Assay was repeated, at least, three times independently and data are represented as ± SEM.

### MIC Assay

We determined bacterial growth inhibition using an adapted version of the broth microdilution method^40^ in which peptides or synbodies were diluted with 1x PBS and added in triplicate wells to bacterial suspensions of ~10^6^ CFU/mL in Mueller-Hinton Broth (MHB) in 96-well polypropylene plates^18,19^. Plates were incubated with shaking overnight at 37 °C and the OD_600_ was measured using a microplate reader. Visible growth was defined as OD < 0.08 or < 10% growth relative to the untreated control. Assays were performed in duplicate.

### Hemolytic Assay

Red blood cell toxicity was measured using a previously published hemolytic assay^18^. These experiments followed an animal use protocol (1099 R) that was reviewed and approved by the Arizona State University Institutional Animal Care and Use Committee an all experiments were performed in accordance with relevant guidelines and regulations. Briefly, blood from female BALB/C mice was collected via terminal bleed, centrifuged at 300 g for 5 minutes, the sera was discarded, red blood cells were rinsed three times with 1xPBS (v/v – RBC: PBS), and centrifuged at 900 g for 15 minutes. Washed RBCs were diluted to 4% in 1x PBS and 50 μL were dispensed into triplicate wells of a 96-well plate. Synbody samples (50 μL), ddH_2_O positive controls, or 1x PBS negative controls were added to each well and the plate was allowed to incubate at 37 °C for 1 hour. The plate was centrifuged at 1000 g for 5 minutes and 90 μL of the supernatant from each well was removed and dispensed into a new 96 well titer plate. The absorbance at 414 nm was measured and the percent hemolysis was calculated using the following formula: percent hemolysis = [Avg. Sample Absorbance/Avg. Control Absorbance] * 100. Assays were performed in duplicate.

## Electronic supplementary material

Supplementary Information
