# Supplementary material for: A Simple Platform for the Rapid Development of Antimicrobials
Source: Sci Rep. 2017 Dec 14;7:17610. doi: 10.1038/s41598-017-17941-7 (PMC5730575; doi:10.1038/s41598-017-17941-7)
Supplement: Supplementary file 1 — Supplementary Information [file 41598_2017_17941_MOESM1_ESM.pdf]

Supplementary Information for:

A Simple Platform for the Rapid Development of Antimicrobials

Stephen Albert Johnston[1,*], Valeriy Domenyuk[1,@], Nidhi Gupta[1,#], Milene Tavares Batista[1], John C. Lainson[1], Zhan-Gong Zhao[1], Joel F. Lusk[1,$], Andrey Loskutov[1,^], Zbigniew Cichacz[1], Phillip Stafford[1], Joseph Barten Legutki[1,+], Chris W. Diehnelt[1]

[1]Biodesign Institute Center for Innovations in Medicine, Arizona State University, Tempe, Arizona 85281, United States

Abstract: Recent infectious outbreaks highlight the need for platform technologies that can be quickly deployed to develop therapeutics needed to contain the outbreak. We present a simple concept for rapid development of new antimicrobials. The goal was to produce in as little as one week thousands of doses of an intervention for a new pathogen. We tested the feasibility of a system based on antimicrobial synbodies. The system involves creating an array of 100 peptides that have been selected for broad capability to bind and/or kill viruses and bacteria. The peptides are pre-screened for low cell toxicity prior to large scale synthesis. Any pathogen is then assayed on the chip to find peptides that bind or kill it. Peptides are combined in pairs as synbodies and further screened for activity and toxicity. The lead synbody can be quickly produced in large scale, with completion of the entire process in one week.

Supplementary Table S1. Microorganisms used in this study.

Organism	Strain	Classification	Inactivation	Screened on		
				10k	275	100 array
Acinetobacter baumannii	ATCC 15149	Gram-negative	None	X	X	X
Bacillus subtilis		Gram-positive	None	X	X	
Burkholderia pseudomallei	ANG-BURK003	Gram-negative	G.	X	X	
Escherichia coli O111:B4	ATCC 12015	Gram-negative	None	X	X	
E. coli O157:H7	ATCC 1883	Gram-negative	None	X	X	X
Francisella tularensis	Schu S4	Gram-negative	G. At.	X	X	
Pseudomonas aeruginosa	PAO1	Gram-negative	None	X	X	
Rickettsia prowazekii	Cairo	Gram-negative	G.	X		
R. prowazekii	Madrid	Gram-negative	G.	X	X	
Staphylococcus aureus	UAB637	Gram-positive	None	X	X	
S. epidermidis	MN8	Gram-positive	None			X
Streptococcus mutans	UAB149	Gram-positive	None	X	X	
S. pneumoniae	STREP5	Gram-positive	None	X	X	
Adenovirus	Adenoid 6	Non-enveloped, dsDNA				X
Cytomegalovirus	AD169	Enveloped, dsDNA	BPL	X	X	
Dengue Virus	Type 2	Enveloped, +ssRNA	BPL	X	X	
Herpes Virus	HSV-2G	Enveloped, dsDNA	Ag.	X	X	
Influenza H1N1	A/PR/8/34	Enveloped, -ssRNA	None	X	X	X
Influenza H1N1	A/CA/07/2009	Enveloped, -ssRNA	None			X
Influenza H3N2	A/Uruguay/716/2007	Enveloped, -ssRNA	At.	X	X	
Influenza B	B/Brisbane/60/2008	Enveloped, -ssRNA	At.	X		
Norovirus	Norwalk GI.1	Non-Enveloped, +ssRNA	VLP	X		
Rotavirus	SA-11	Non-enveloped, dsRNA				X

Rubella Virus	HPV77	Enveloped, +ssRNA	UV	X	X	X
SARS Coronavirus	Urbani	Enveloped, +ssRNA	UV	X	X	
Vaccinia Virus	Lister	Enveloped, dsDNA	P+UV	X	X	
Varicella Zoster Virus	ROD	Enveloped, dsDNA	UV?	X	X	
Venezuelan Equine Encephalitis Virus	Trinidad 1A/B	Enveloped, +ssRNA	BPL + G			

BPL = b-propiolactone inactivation. UV = UV inactivation. P+UV = psoralen + UV inactivation. G = gamma irridation. At. = attenuated organism. Ag. = surface antigen. VLP = virus-like particle.

Supplementary Table S2. Peptides sequences of 100 peptide library.

Peptide Number	Peptide Sequence	Peptide Number	Peptide Sequence
2	CSGCRRAGRPLLVCHPAHGG	123	CSGHLMHIHYWHKPGTPYPM
3	CSGDFLSHKDKKMHKWKWDE	124	CSGHLNTRHLFHGFQPIMHP
5	CSGDMYEYNPFQGNHIYNKK	125	CSGHNIYAQYGYPYDHMYEG
8	CSGEGWYRDEAPAQLNDKMK	127	CSGHYADDKKNMTEKYKMHY
20	CSGLGKNQAKHLHGTYDSKI	128	CSGHYGSAFDKEMDTKGHRA
23	CSGNSDYNAIWQKHVEAHKE	129	CSGHYNRQYNNYYQGKYRSL
36	CSGVGWHKSLQQQGPELPPQ	134	CSGKDHNAQDQESVHWKYKG
38	CSGWALRIREHADIDNYVKR	137	CSGKKHGPKYYYMSDRVVNQ
40	CSGYADQYMYKQSPMYPKTD	145	CSGKRRKQANPRRKNALESE
43	CSGYNWSRKKHKYYPRKIAY	147	CSGKRYLQKGKGALRGLYIF
45	CSGYVINGIMTSGAMAGSHK	149	CSGKSQEIGDPDDIWNQMKW
47	CSGAEVYLQAHFEGWASKAK	150	CSGKTEHYMPNNNTFGYEYE
50	CSGALTVHKQCHKLGGTVLP	151	CSGKVHRWKYHSRQNYTYRP
52	CSGAMHGPATTRPAIGRYPW	155	CSGLFPVKTWKYWKKAYSIA
53	CSGANPWEKEDDRRYSDYKH	156	CSGLIPKRAEYKQHFMQHRT
54	CSGAQEWAAKSYKWNKDGYL	157	CSGLLHELPDDYEKINPQKY
58	CSGAVNYQEVFNKARMKKKR	158	CSGLLYHFKVGLRTMKISMM
63	CSGDEFMGHAFGHKLQAIKN	163	CSGMCQMYQPWMCKKYYRLL
67	CSGDLPPSEHASMPDVRKKQ	165	CSGMIHYHMGYQKKYDTSNH
68	CSGDMHWGYQDGKTLVPTSK	166	CSGMKQPKHNKINDNPKAYE
70	CSGDNQGFNKMYPKSMGFVH	169	CSGMVIHDVAPKQPKPHGWS
71	CSGDPTHATEPKRYEAYNDH	170	CSGMWRHSKKKEDPYDLKNW
72	CSGDPVQLIHPMDWEGQKYH	172	CSGNDKKGNYKLLYASNAYK
73	CSGDRENDKSFQKRKDAGVI	174	CSGNETAPDNTYRYKQSAQK
74	CSGDSKSIHIMPVHMIFPPD	176	CSGNKVHPKSKEQKVYINYP
75	CSGDSPMGYYHQKTSPWADK	179	CSGNPNTWQINYPHLTYHRA
79	CSGDVAQFTSSGMYRPANKM	181	CSGNQKTDRKHKKYQWWEIY
83	CSGEDSDEPYWPQPPKHWKH	183	CSGNWGSEWKKVKAPHNQKP
84	CSGEKMMTKYFFEKYGAAMP	186	CSGPENEMKSIVIFPEKKDH
85	CSGELFFTRDNNMHNHMHKM	189	CSGPGQQWIGNDWKAVQKGK
86	CSGELYSPRMKAIYSYHWKH	190	CSGPHFMFEPSVVRPNYKQA
88	CSGEMWAIMPPIIKPDNKGH	192	CSGPHPQYRHGPKHYIRTQM
91	CSGEPSMQKYKLKGGKLNEH	204	CSGQFSAKKYWEIKPMDYWK
93	CSGEWPDIKHKYNMNQFVSR	206	CSGQITSHQKYKSVFNQHMG
94	CSGEWRQQKMMPKSAPNKPQ	207	CSGQPESNHGRKMSMIYPKE
95	CSGEYMLKTEPHEDHRDKYW	210	CSGQYSQQSSSYGQQMFKKE
96	CSGFKDFDDDYFNVSKKYIW	216	CSGRPHAHVERMKTDRQYAW
98	CSGFKNYDMVEDKHNKHAYA	219	CSGRQSKRYKEFGKDPTKAH
101	CSGFMPKVKMWFEEMVQQHK	223	CSGSAFDQKDSADASKWGYK
104	CSGFRKHPWKHPRHKFHKPW	227	CSGSFNQEYFPYPMIDYLKK
105	CSGFRKYDIHAMYKPRNKYQ	228	CSGYYDVHMGPMNDNHYFKK
107	CSGFWPRKHPFRWKHPRPKH	233	CSGSLVWNNGDYKYNPKMPS
110	CSGGAKSKEKDGYQVPLSHS	241	CSGTANELLYYKNYGVKNPK
111	CSGGAVNKYHYDYKTKKKPY	247	CSGTTVEPTPGKGPKVYRRK
113	CSGGKDSSQDAYSMKQMIRS	252	CSGVDSNSKYEIGKEHDLKK
114	CSGGKGPKVYRRKHQELQAM	256	CSGVLQTVHGPEAVGLSKVK
116	CSGGSLTAIKTRAPQELKFL	263	CSGWLMSVIKADRKAHRKEQ
119	CSGHELEEAKKPPSQRWEHK	268	CSGYGGYHEQFGMMEHPSSK
121	CSGHKFNFHLNEHAHWLSGR	272	CSGYLDRKELGRCAQAQMNK
122	CSGHKQCHKLGGTVLPESFC	274	CSGYYNVHRPFIDPARDHPM

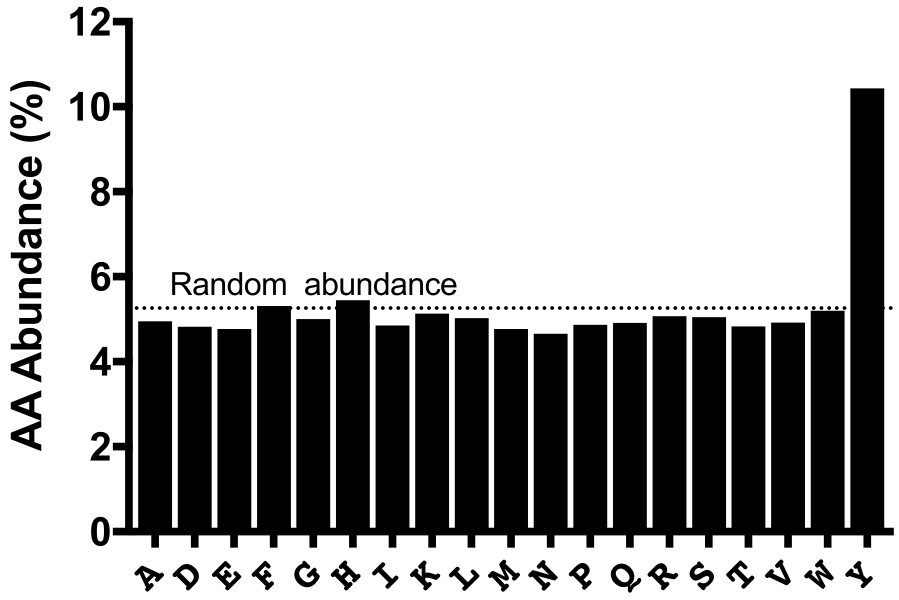

Supplementary Figure S1. Amino acid abundance for the peptides in the 10,000 peptide microarray. The dashed line indicates the percent abundance if each amino acid was of random distribution.

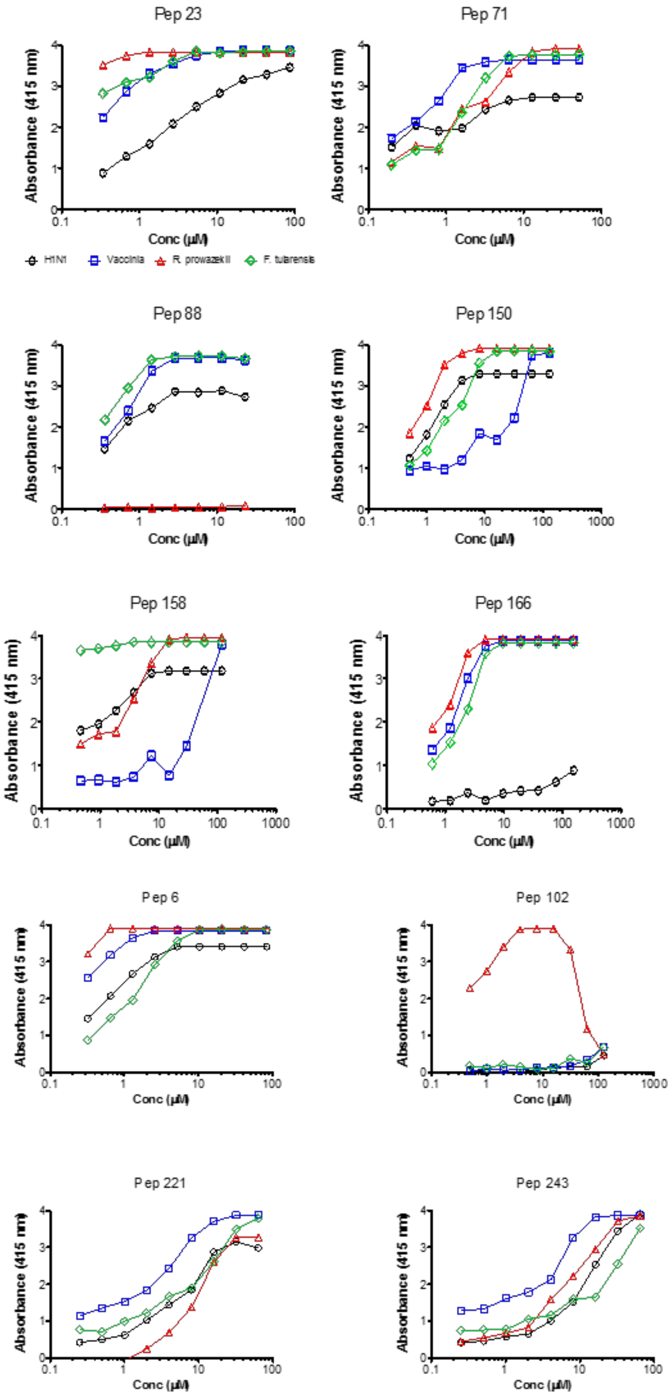

Supplementary Figure S2. ELISA screening for the 275 peptides library. Four pathogens (Vaccinia virus, Influenza virus A/PR/8/34 (H1N1), *F. tularensis* or *R. prowazekii* Madrid) were tested against the 275 peptides sequences to identify those with broad binding spectrum by ELISA.

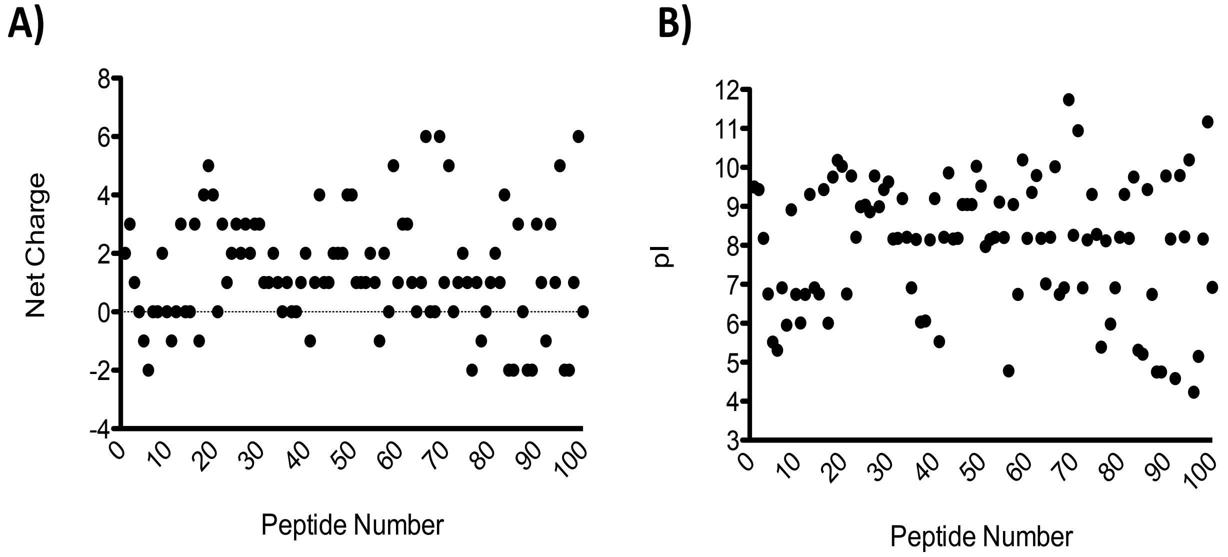

Supplementary Figure S3. Properties of 100 peptide library. Distribution of **A)** net charge and **B)** pI of peptide library.

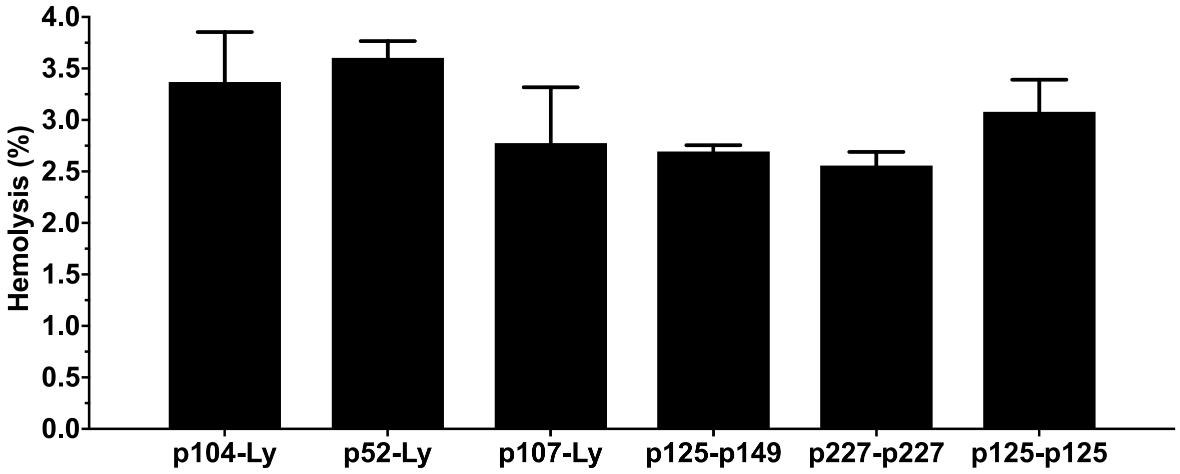

Supplementary Figure S4. Evaluation of hemolysis by the pathogen-specific synbodies. The synbodies selected (125 μM) against Influenza virus (p125-p149, p227-p227) and S. epidermidis (p104-Ly, p52-Ly, p107-Ly) were incubated with mice red blood cells and hemolysis was measured after 60 minutes.
